# Functional improvement by behavioural activation for depressed older adults

**DOI:** 10.1192/j.eurpsy.2023.2433

**Published:** 2023-07-27

**Authors:** Noortje P. Janssen, Richard C. Oude Voshaar, Sanne Wassink-Vossen, Gert-Jan Hendriks

**Affiliations:** 1 Behavioural Science Institute, Radboud University, Nijmegen, The Netherlands; 2 Department of Primary and Community Care, Research Institute of Health Sciences, Radboud University Medical Centre Nijmegen, Nijmegen, The Netherlands; 3 Institute for Integrated Mental Health Care Pro Persona, Nijmegen, The Netherlands; 4 Department of Psychiatry, University of Groningen, University Medical Centre Groningen, Groningen, The Netherlands; 5 Department of Old Age Psychiatry, GGNet Mental Health, Warnsveld, The Netherlands

**Keywords:** behavioural activation, functional impairment, older adults, primary health care

## Abstract

**Background:**

Recovery in mental health care comprises more than symptomatic improvement, but preliminary evidence suggests that only collaborative care may improve functioning of depressed older adults. This study therefore evaluates the effectiveness of behavioural activation (BA) on functional limitations in depressed older adults in primary care.

**Methods:**

This study uses data from a multicentre cluster randomised controlled trial in which 59 primary care centres (PCCs) were randomised to BA and treatment as usual (TAU), and 161 consenting older (≥65 years) adults with clinically relevant symptoms of depression participated. Interventions were an eight-week individual BA programme by a mental health nurse (MHN) and unrestricted TAU. The outcome was self-reported functional limitations (WHODAS 2.0) at post-treatment (9 weeks) and at 12-month follow-up.

**Results:**

At the end of treatment, the BA participants reported significantly fewer functional limitations than TAU participants (WHODAS 2.0 difference −3.62, *p* = 0.01, between-group effect size = 0.39; 95% CI = 0.09–0.69). This medium effect size decreases during follow-up resulting in a small and non-significant effect at the 12-month follow-up (WHODAS 2.0 difference = −2.22, *p* = 0.14, between-group effect size = 0.24; 95% CI = -0.08–0.56). MoCA score moderated these results, indicating that the between-group differences were merely driven by those with no cognitive impairment.

**Conclusions:**

Compared to TAU, BA leads to a faster improvement of functional limitations in depressed older adults with no signs of cognitive decline. Replication of these findings in confirmatory research is needed.

## Introduction

Depression is the most common psychiatric illness worldwide, that can impair functioning and is related to increased morbidity and mortality in older adults [1, 2]. Functioning describes how well people are able to participate in different domains of life, such as mobility, cognition, and life activities [3]. Depression and ageing are both related to functional decline [4–6]. Most studies investigating the treatment of late-life depression focus on symptomatic improvement but not functional improvement. Older adults value functioning more than longevity and consider improved functioning as an important factor in remission from depression [3, 7]. If we fail to investigate how treatments influence functioning, we lack important information regarding their health benefits for older adults. In a prospective cohort study of older adults with depression, only 30.5% of remitted older patients achieved functional recovery [8]. Psychotherapeutic interventions such as behavioural activation (BA) might positively influence functioning because they catalyse activity engagement by encouraging people to move their bodies and interact with the world. By being increasingly active, people potentially strengthen their skills, such as attention, balance, and physical endurance, which may translate into fewer functional limitations.

BA is a short and effective low-threshold therapy that can be easily implemented in primary care [9]. It can be effectively delivered by non-specialised health practitioners such as mental health nurses [10]. Within BA, the therapist and patient work together to create a personal environment of positive reinforcement by increasing functional and pleasurable behaviour, and by decreasing avoidant and depressed behaviour such as irregular sleeping and eating patterns and avoidance [11]. BA is as effective as cognitive behavioural therapy (CBT) and antidepressant medication, and more effective than control conditions in reducing depression in adults [12, 13]. Small studies show promising effects for older adults as well [14]. A recent meta-analysis showed that psychological interventions in general show mixed results regarding their impact on functional recovery in late-life depression [6]. Multicomponent treatments, such as collaborative care, are the most promising in improving functioning in older adults with depression [6]. Collaborative care usually requires a multi‐professional approach, a structured management plan, scheduled patient follow-ups, and enhanced communication between professionals [15]. Implementing a collaborative care system is more complex and possibly costlier than implementing BA in primary care. Therefore, it’s useful to investigate whether BA, a simple low-threshold treatment, results in functional improvement for depressed older adults. However, until now little research has been conducted about the effects of BA on functioning [16].

In a recently conducted cluster randomised trial, we showed that among depressed older adults, BA led to a faster reduction in depressive symptoms than treatment as usual (TAU) [17]. Using the data of that trial, in the current study we aim to investigate how BA impacts functioning in depressed older adults. We hypothesise that BA leads to more functional improvement than treatment as usual in primary care.

## Methods

### Design

This study was conducted in the context of a cluster randomised trial in primary care that investigated the effectiveness of BA as compared to TAU as a treatment for older adults with clinically relevant depressive symptoms [18]. In this study, 59 primary care centres (PCCs) were randomised. In half of the PCCs, mental health nurses (MHNs) were trained to deliver an 8-week BA protocol, and in the other half, PCCs delivered treatment as usual. A total of 161 participants were recruited for the study. Results showed that BA lead to better depressive outcomes at post-treatment and 3-month follow-up, but not in subsequent follow-ups (6–12 months after treatment ended) [17]. Primary outcome measurements were completed at baseline, at weeks 2, 4, and 7 during treatment, at post-treatment, and at subsequent 3-, 9-, and 12-month follow-up, but functional limitations (as a secondary outcome of the RCT) only at baseline, post-treatment (9 weeks), and the 12-month follow-up. Details about the study protocol and results can be found elsewhere [17, 18].

### Participants

Participants were older adults (≥65 years) who presented with depressive symptoms at their general practice either spontaneously or after reading an information leaflet about the ongoing trial. Inclusion criteria were (a) current clinically relevant depressive symptoms as measured with the Patient Health Questionnaire 9 (PHQ-9 ≥ 10) [19] and (b) age 65 years or older. Exclusion criteria were (a) current severe mental illness, high risk of suicide, drug and/or alcohol abuse in need of specialised treatment as assessed with the Mini International Neuropsychiatric Interview (MINI5.0.0) [20]. In case the MINI results suggested a severe mental condition, an old age psychiatrist as well as the patients’ GP were consulted to prevent iatrogenic damage by study participation. Only patients in need of specialised care were not eligible. Other exclusion criteria were (b) undergoing psychotherapy in the previous 12 weeks or current treatment by a mental health specialist or (c) having moderate to severe cognitive impairment, as measured with the Montreal Cognitive Assessment (MoCA < 18) [21]. Physical illnesses, comorbid psychological disorders that were not in need of specialised treatment, disabilities, or mild cognitive impairments were not exclusion criteria, neither were illiteracy or nonperfect understanding of the Dutch language, as we offered assistance with filling out the questionnaires at home in several languages by independent, blinded research assistants. Patients with antidepressants were eligible provided that a stable dose had been maintained for at least 12 weeks before participating in the study.

### Outcomes

#### Functional limitations

The World Health Organization Disability Assessment Schedule 2.0 (WHODAS 2.0, short version) was used to evaluate functional limitations. The WHODAS assesses the level of functioning in six domains of life (i.e., cognition, mobility, self-care, getting along, life activities, and participation) [22]. The Questionnaire was administered at baseline, end of treatment, and 12-month follow-up. The short version consists of 12 items on a 5-point Likert scale, can be completed in less than 5 minutes [22], and is a reliable and valid self-report instrument to assess functioning [23]. A simple scoring method was used, which used the sum score of the 12 items. This “simple” scoring is straightforward and can be directly applied in primary care. Moreover, normative data are available for this scoring method, facilitating comparisons with other populations [24]. This simple score ranges from 0 (no functional limitations) to 48 (severe functional limitations).

#### Physical illness

To determine whether physical illness at baseline moderated the results, we used the number of medicines used for physical conditions prescribed by a physician as a proxy, as done in other studies (e.g., [25]). A higher number of medicines has been associated with worse depression outcomes in older adults [26]. We derived this information from a modified version of the Trimbos and Imta self-report questionnaire on Costs associated with Psychiatric illness (TiC-P) with a recall period of 3 months. The TiC-P is a widely used instrument to measure healthcare utilisation and lost productivity [27]. In one of the questions of this instrument, participants wrote down which prescribed medications they had used in the last 3 months. We excluded psychotropic drugs, such as antidepressants, antipsychotics, and tranquilisers, and used the sum of all other medications as a proxy for physical illness at baseline.

#### Cognitive functioning

The Montreal Cognitive Assessment Scale was used to test whether baseline cognitive functioning predicted or moderated treatment results. The MoCA is a short cognitive screening tool that tests a wide range of cognitive functions [21]. The tool has a high sensitivity as well as specificity for detecting mild cognitive impairment, and includes executive functioning [21]. A cut-off score of ≤25 is indicative of mild cognitive impairment in healthy older adults [21]. In the current study, all participants had a MoCA score of 18 or higher to preclude non-demented patients due to interference of depression with cognitive functioning.

### Statistical analyses

The baseline characteristics of the participants and the PCCs were summarised using the means for continuous data and percentages for categorical data. We tested for baseline differences to ensure that cluster randomisation had led to comparable treatment groups. We used the R software version 4.1.1 [28] within Rstudio version 2021.09.0 + 351a, specifically the lme4 and lmerTest packages [29], to model the differences in the course of functional improvement between BA and TAU with linear mixed-effects models. Prior to entering treatment effects in the models, we fitted two models to examine whether the PCC level added additional predictive power to the model (see Supplementary material S1). To model the effect of treatment on functioning over time, while accounting for the intra-subject correlation between the different time points, we used flexible functions known as regression splines, including pre-treatment assessment of depression as a covariate and the random part of the model with one internal knot placed at the 9-week mark of end of treatment. We modelled the effect of time using linear and quadratic splines and selected the model with the best fit based on the Akaike information criterion values (AICs) and Bayesian information criteria (BICs) [30]. Fixed effects were decomposed by ANOVA and evaluated by the F-statistic. We computed Estimated Marginal Means (EMMs) using the R emmeans package [31] and examined whether the differences between the groups were significant at post-treatment and 12 months after post-treatment, by performing t-tests on these EMMs and computing standardised between and within effect sizes (Cohen’s *d*). For the effect size calculation, we used the SD of the complete sample at baseline.

The intention-to-treat analyses included all participants, regardless of the number of sessions received. The use of mixed models with time, treatment, interaction between time and treatment, and baseline covariate as parameters obviates separate multiple imputation methods for missing data [32].

### Sensitivity analyses

We added age, physical illness at baseline, education, cognitive impairment, and sex as covariates in the model to explore whether these factors predicted or moderated outcomes of the main analysis. Age at baseline was added as a continuous variable. Sex was added as a dichotomous variable indicating whether someone was male or female. Education was added as a continuous variable ranging from 1 (no to primary education) to 6 (university degree). MoCA was added as a continuous variable in the model. To plot the effect of MoCA on WHODAS scores over time, we dichotomised between mild cognitive impairment (MoCA = 18–25) or no cognitive impairment (MoCA ≥ 26).

## Results

Baseline characteristics are shown in Table 1. Participants had a mean age of 75.2 (SD = 7.0) and were predominantly female (60.2%). The mean WHODAS 2.0 score at baseline was 16.9 (SD = 9.3). There were no significant baseline differences between groups. Treatment as usual consisted mostly of eclectic counselling by mental health nurses (50.6%) and antidepressants (14.3%).

**Table 1.** Baseline information

Patient characteristics	BA (*n* = 96)	TAU (*n* = 65)	*p*-value^a^
Age in years (SD)	75.8 (6.8)	74.3 (6.0)	0.14
Sex			
Female	61 (63.5%)	36 (55.4%)	0.3
Marital status			
Living alone	48 (53%)	37 (59%)	0.5
Level of education			0.5
Low	39 (42.4%)	27 (42.2%)	
Intermediate	28(30.4%)	28 (43.8%)	
High	25 (27.2%)	9 (14.1%)	
Duration of current depression^b^ in years (SD)	8.6 (15.8)	11.4 (17.7)	0.3
Baseline functional limitations, WHODAS 2.0, (SD)	17.25 (9.63)	16.38 (8.90)	0.6
Number of medicines^c^ (SD)	4.8 (3.0)	4.9 (2.9)	0.4
Current depressive disorder	50 (52.1%)	36 (55.4%)	0.7
Baseline depression, QIDS-SR (SD)	13.4 (4.1)	12.7 (3.7)	0.3
Cognitive impairment, MoCA^d^ ^,^^e^			0.3
Mild cognitive impairment (18–25)	48 (51.1%)	22 (36.1%)	
No cognitive impairment (26–30)	46 (47.9%)	39 (63.9%)	

a One-way ANOVA for continuous variables; Pearsons’s Chi-squared test for dichotomous variables.

b Depression is “clinically relevant depressive symptoms” (PHQ-9 > 9).

c number of medicines used for physical conditions prescribed by a doctor.

d Montreal cognitive assessment.

e MoCA scores are missing for 5 participants that were included during a COVID-lockdown.During this time period, GPs only referred patients that certainly did not have cognitive decline.

Note. percentages are based on totals of available data.

## Main results

Available data from all but one of the participants (*n* = 160) were used in the intention-to-treat model. The missing participant had not filled out any questionnaires after completing the informed consent. The best-fitting model used linear splines with a knot at post-treatment (9 weeks). The comparison between different models can be found in Supplementary Table S1.

**Figure 1.**
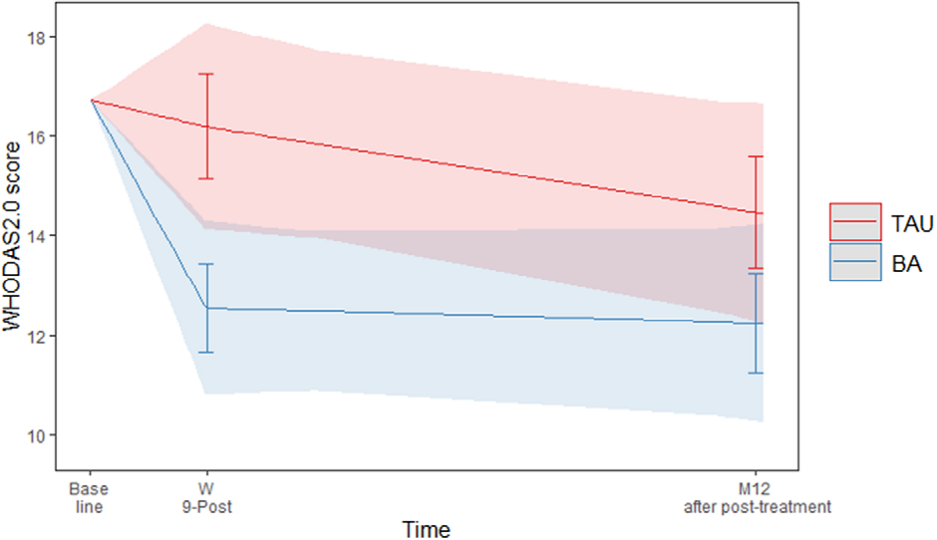
Predicted values of WHODAS 2.0 with 95% confidence interval and error bars.

The interaction between treatment arm and functioning over time was significant (F(2, 108) = 3.52, *p* < 0.05). Figure 1 shows the predicted values of WHODAS 2.0. At the end of treatment (9 weeks), BA participants reported significantly better functioning than TAU participants (EMM difference of WHODAS2.0 = −3.62, *p* = 0.01) but not in the follow-up at 12 months (EMM difference of WHODAS2.0 = −2.22, *p* = 0.14).

The between-groups effect size for the WHODAS at the end of treatment was d = 0.39 (95% CI 0.09–0.68), a small to medium effect [33]. At the 12-month follow-up, the between-groups effect size was small, but not significant (d = 0.24 (95% CI–0.08–0.56)) [34]. All EMMs and effect sizes can be found in Table 2.

**Table 2.** Tests of WHODAS2.0 EMMs

	Treatment group (Estimated marginal mean (SE))				Estimated treatment difference		
Assessment^a^	BA	ES^b^	TAU	ES^b^	Difference	ES^c^ (95% CI)	*p*-value
Post-treatment^d^	12.54 (1.38)	0.44	16.18 (1.38)	0.06	−3.62	0.39 (0.09–0.68)	0.01
Follow-up 12M^e^	12.24 (1.51)	0.48	14.46 (1.51)	0.24	−2.22	0.24 (−0.08–0.56)	0.14

a Baseline differences in EMMs are not shown because the model corrects for differences.

b ES, within-group effect size, difference from baseline WHODAS2.0 score per measurement point.

c ES, between-group effect size.

d Post-treatment is at 9 weeks after baseline.

e Follow up 12 M months is 12 months after post-treatment.

### Sensitivity analyses

**Figure 2.**
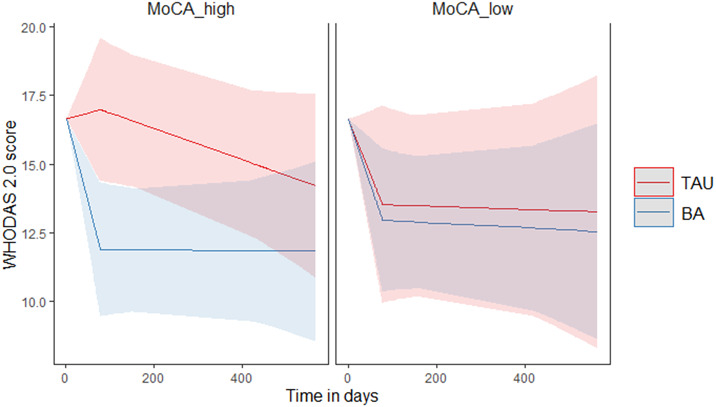
Predicted values of WHODAS 2.0 sorted by MoCA score. (A) WHODAS values for participants with no cognitive impairment (MoCA > 25). (B) WHODAS values for participants with mild cognitive impairment (MoCA = 18–25).

Age, physical illness at baseline, education, and sex did neither moderate nor predict the results. There was a significant moderating effect of MoCA on functioning (F(2,101) = 4.95, *p* = 0.02). For participants with a high MoCA score (≥26), indicating no cognitive decline, BA treatment was significantly better than TAU (EMM difference = −4.10, *p* = 0.006). BA had a clear treatment effect that was maintained during follow-up. TAU resulted in a smaller effect, which was found between end of treatment and 1-year follow-up. For participants with a low MoCA score (18–25), both TAU and BA were effective, showing a decrease in WHODAS score during treatment that was maintained during follow-up (Figure 2). Detailed results of these sensitivity analyses can be found in chapter 2 of the Supplementary material.

## Discussion

To our knowledge, this is the first study that has examined the impact of face-to-face BA on functioning in depressed older adults. Our results show that, within BA, functioning improved significantly at post-treatment and that this improvement remained until the 12-month follow-up. When compared to TAU, we demonstrated that BA participants show significantly greater improvement at post-treatment, but lost significance at the 12-month follow-up, due to further improvement among patients receiving TAU. Participants in the TAU condition commonly received continued care well beyond the post-treatment mark, while treatment of participants in the BA condition ended around the post-treatment mark, but their levels of functional improvement remained stable in the subsequent year of follow-up.

Unfortunately, norm scores for the WHODAS 2.0, indicating the severity of functional limitations, are not available yet. However, compared to a sample of 65- to 85-year-old people with at least one mental disorder, the baseline level of functional limitations of participants in this study was considered high [24]. The average baseline score in our sample was between the 85th and 90th percentile, indicating more functional limitations than the aforementioned norm group. At post-treatment, the scores of the BA group fell between the 50th and the 75th percentile, while the scores in the TAU group dropped to just below the 85th percentile [24]. Normative data regarding clinically meaningful change for depressed older adults are lacking as well. Studies in different populations have found difference scores between 5% (<2.5 points) and 9 points to be clinically meaningful [35–37]. More data and validation of the WHODAS 2.0 in psychiatric older samples are needed to conclude whether the observed difference of 5 points in a depressed older population can be considered clinically relevant.

Sensitivity analyses showed that cognitive impairment moderated the outcomes. The functioning of participants without cognitive impairment improved significantly in BA than in TAU during treatment. For participants with mild cognitive impairment, there was no statistical difference between the effectiveness of BA and TAU. This effect may be explained by the origin of functional impairment in both groups. Functional impairment in the group without cognitive impairment may be mainly caused by depression, and therefore best treated with a focused, short depression-specific treatment such as BA, whereas functional impairment in a cognitively impaired group might be partially explained as a consequence of the cognitive impairment itself [38]. Therefore, the effectiveness of BA and TAU does not significantly differ regarding improvements in functional impairment in the latter group. It has to be noted however that this moderating effect of MoCA was only present with regard to functional improvement and not with regard to depressive symptoms. Our previous study showed that BA led to a faster decline in depressive symptoms regardless of MoCA score [17]. Therefore, the benefits of BA seem to outweigh TAU, even in a mildly cognitively impaired group.

Several studies have shown that healthcare practitioners can be reluctant to prescribe psychotherapy to older adults [39]. One study found that psychologists expected older patients to have a poorer prognosis and be less appropriate for therapy than their younger counterparts [40]. Furthermore, a study showed that older women experience more “benevolent sexism,” which means that physicians tend to be more protective and thereby a priori may have low expectations of goal-oriented, directive psychotherapies with a focus on behavioural change and reduction of symptoms in case older patients [41, 42]. The current study shows that these factors, with the exception of cognitive impairment, do not influence the treatment results of depressed older adults. Age, sex, education, and physical health neither predicted functional improvement nor confounded our findings. These results show that the concerns that general practitioners might have about a possible lack of feasibility and effectiveness for BA for older frail patients are not confirmed by this study. We showed that functioning improved in both treatment groups, regardless of the abovementioned factors, with a larger effect size in the BA group. We found similar effects regarding the lack of influence of these potential confounders on depressive symptoms [17].

These results have some clinical implications. First, GPs should not use age, sex, and education as a means to determine whether BA would be indicated for functional improvement. Our study showed that functioning improved more in BA than in TAU regardless of these factors. Second, cognitive impairment did moderate treatment results. When GPs suspect cognitive problems, a MoCA score under 26 might indicate that BA and TAU are equally effective, regarding functional improvement, whereas participants with a MoCA score of 26 or higher improve more and faster with BA. Choosing a short-term treatment such as BA for this population reduces healthcare costs while accelerating functional and clinical improvement.

One previous study investigated the effect of BA on functioning in older adults [16]. This randomised controlled trial showed that tele-delivered problem-solving therapy (PST) by licensed clinicians and tele-BA by lay counsellors were significantly better at reducing functional disability than attention control (AC). Compared to AC, the tele-BA group showed an effect size of 0.43 (95% CI, 0.21 to 0.65), which is similar to the results of our study. A true comparison is not possible, however, because TAU in the current study was an active control group consisting mostly of counselling and medication. Depression in older adults is usually complex, and while antidepressant medication may (partially) alleviate depressive symptoms, it does not act on predisposing and precipitating factors such as the social context, multimorbidity, and physical and cognitive decline. BA does take into account such factors by focusing on increasing healthy behaviours that in turn increase both physical and mental well-being [11]. A recent meta-analysis showed mixed results for psychological interventions and suggested a collaborative care approach in primary care. However, our data seem to suggest that a standalone BA approach, well embedded in primary care, might be effective as well in improving functioning [6]. Collaborative care comes with higher costs than the implementation of a standalone therapy by non-specialists in primary care.

This study has several strengths. First, the ecological validity of the study is high. We lowered the threshold for participation by visiting all patients at home and offering at-home assistance with questionnaires. Therefore, we were able to investigate a relatively old and frail group of home-dwelling older adults. Second, we used a state-of-the-art mixed models design which allowed us to investigate the course of symptoms over time. Third, we investigated several important confounders that could have potentially influenced the results.

The study also has some limitations. First, while designing the study, functioning was included as a secondary outcome parameter for which the study was not powered. Therefore, the results are exploratory and need to be confirmed by confirmatory research. Second, most of the participants were Caucasian. We do not know how these results generalise among other ethnic groups. Third, we used the self-reported number of medicines prescribed by a doctor for physical illness as a proxy for physical health, but research about the validity of this measure is scarce. Participants might have over- or underreported used medication. However, research shows that older adults do not always consistently use medication as prescribed [43] and that self-report is a valid tool to register used medication [44]. Relying on self-report instead of medical records enabled participants to record the medication actually used, rather than prescribed. Furthermore, the number of medicine as a proxy for physical health seems to be a promising measure, outperforming other measures such as the number of chronic illnesses [25].

## Conclusions

BA, intended to alleviate depressive symptoms, effectively improved functioning in depressed older adults in primary care at post-treatment but not at the 12-month follow-up. Participants without cognitive impairment, regardless of age, sex, or level of education, showed faster functional improvement in BA compared to TAU. Future research should investigate the association between specific cognitive domains and functional improvement. The implementation of BA in primary care might increase accessibility of psychotherapy, and thereby improve depressive symptoms as well as functioning in older adults. Replication of these findings in confirmatory research is needed.

## Supporting information

Janssen et al. supplementary material Janssen et al. supplementary material

Janssen et al. supplementary material Janssen et al. supplementary material

## Data Availability

The data will be available at DOI https://doi.org/10.34973/1dk9-tj89, after completion of the project, but no later than December 2025, due to ethical considerations, which include ensuring that the release of the data does not interfere with the ongoing analyses of the research group. Until that time, researchers can obtain the data through consultation with the corresponding author.

## Supplementary material

The supplementary material for this article can be found at https://doi.org/10.1192/j.eurpsy.2023.2433.
