# Supplementary material for: Functional improvement by behavioural activation for depressed older adults
Source: Eur Psychiatry. 2023 Jul 27;66(1):e62. doi: 10.1192/j.eurpsy.2023.2433 (PMC10594265; doi:10.1192/j.eurpsy.2023.2433)

Supplementary Materials: The effectiveness of Behavioural activation on functional limitations for depressed older adults in primary care: Secondary results of a cluster-randomised trial in primary care

Noortje P Janssen, Richard C Oude Voshaar, Sanne Wassink, Gert-Jan Hendriks

2022-11-07

1 The effectiveness of BA on functional limitations

1.1 Compare models

LM1 = linear model with no knots LMc1 = linear with knot at post treatment LMC2 = quadratic with no knots

Lineair with knot on post treatment has the best fit.

	npar	AIC	BIC	logLik	deviance	Chisq	Df	Pr(>Chisq)
	<dbl>	<dbl>	<dbl>	<dbl>	<dbl>	<dbl>	<dbl>	<dbl>
LM1	7	2298.510	2325.979	-1142.2548	2284.510	NA	NA	NA
LM1c1	11	1191.015	1234.181	-584.5073	1169.015	1115.495	4	3.315437e-240

2 rows

	npar	AIC	BIC	logLik	deviance	Chisq	Df	Pr(>Chisq)
	<dbl>	<dbl>	<dbl>	<dbl>	<dbl>	<dbl>	<dbl>	<lgl>
LM1c1	11	1191.015	1234.181	-584.5073	1169.015	NA	NA	NA
LM1c2	11	1225.222	1268.388	-601.6108	1203.222	0	0	NA

2 rows

1.2 Report best fitting model

Model shows a significant difference between groups at the first part of the model (0-9 weeks), similar to the depression model.

Linear mixed model fit by REML. t-tests use Satterthwaite's method

y ~ bs(dy, knots = 78, degree = 1) * Group + y0 + (bs(dy, knots = 78, degree = 1) + 0 | sid)

Number of obs: 374, nsid, 160

Fixed effects Anova

Coef	Sum Sq	Mean Sq	NumDF	DenDF	F value	Pr(>F)	sig
bs(dy, knots = 78, degree = 1)	0.230	0.115	2	108	11.3	0.000	*
Group	0.00358	0.00358	1	154	0.351	0.554	
y0	13,500	13,500	1	154	1,330,000	0.000	*
bs(dy, knots = 78, degree = 1):Group	0.0719	0.0360	2	108	3.52	0.033	*

* p <= 0.05

Fixed effects:

Coef	Estimate	Std. Error	df	t value	Pr(>|t|)	sig
(Intercept)	0.00742	0.0190	154	0.390	0.697	
bs(dy, knots = 78, degree = 1)1	−0.527	1.05	127	−0.500	0.618	
bs(dy, knots = 78, degree = 1)2	−2.82	1.37	88.3	−2.06	0.042	*
GroupBA	−0.00975	0.0165	154	−0.593	0.554	
y0	1.00	0.000869	154	1,150	0.000	*
bs(dy, knots = 78, degree = 1)1:GroupBA	−3.63	1.38	128	−2.63	0.010	*
bs(dy, knots = 78, degree = 1)2:GroupBA	−1.74	1.85	88.8	−0.939	0.350	

* p <= 0.05

Random effects

Group	Name	Variance	Std.Dev.
sid	bs(dy, knots = 78, degree = 1)1	59.98683	7.74512
sid	bs(dy, knots = 78, degree = 1)2	76.47390	8.74494
Residual	NA	0.01021	0.10103

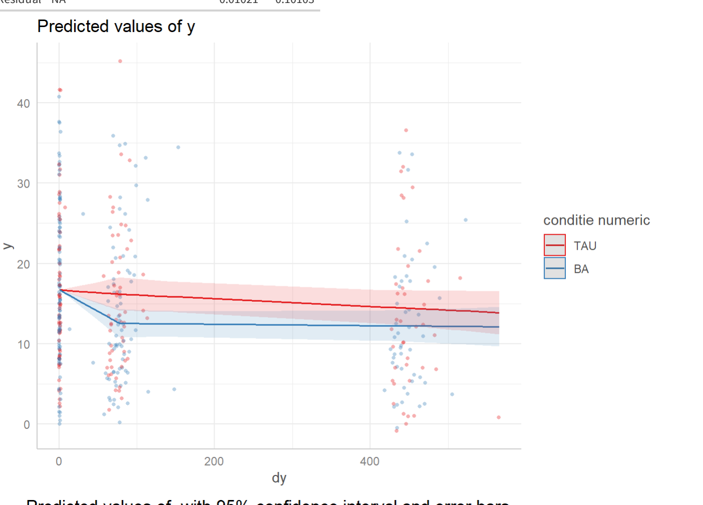

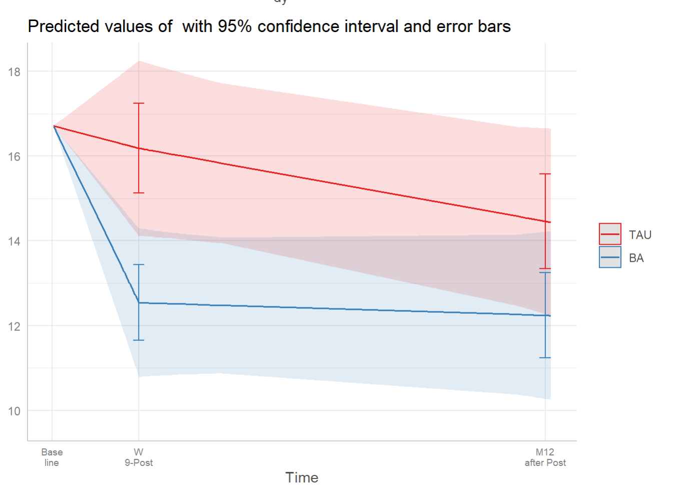

1.3 Calculate EMMs

BA significantly better functioning at post treatment as compared to TAU. Not at 12m FU.

Tests of EMM's whodasS_Ftot

dy	TAU	BA	y0	estimate	SE	df	t.ratio	p.value
0	16.72	16.75	16.70	−0.04	0.02	273.63	−1.53	0.13
78	16.18	12.54	16.70	3.64	1.38	127.35	2.64	0.01
444	14.46	12.24	16.70	2.22	1.51	96.95	1.47	0.14

y ~ bs(dy, knots = 78, degree = 1) * Group + y0 + (bs(dy, knots = 78, degree = 1) + 0 | sid)

1.3.1 Within group effect sizes

We looked at the effect sizes within groups by contrasting the mean WHODAS-scores per group at post-treatment and 12-month follow-up. Used sigma=9.326677 (SD of baseline WHODAS-score)

```
## Linear mixed model fit by REML ['lmerModLmerTest']
## Formula: y ~ bs(dy, knots = 78, degree = 1) * Group + (bs(dy, knots = 78,
##     degree = 1) + 0 | sid)
##    Data: d.lmerdata
## REML criterion at convergence: 2684.351
## Random effects:
##  Groups   Name                            Std.Dev. Corr
##  sid      bs(dy, knots = 78, degree = 1)1 5.955
##           bs(dy, knots = 78, degree = 1)2 5.745    1.00
##  Residual                                 8.126
## Number of obs: 374, groups:  sid, 160
## Fixed Effects:
##                              (Intercept)
##                                  16.2771
##          bs(dy, knots = 78, degree = 1)1
##                                  -1.2964
##          bs(dy, knots = 78, degree = 1)2
##                                  -3.7438
##                                  GroupBA
##                                   0.8354
## bs(dy, knots = 78, degree = 1)1:GroupBA
##                                  -2.2292
## bs(dy, knots = 78, degree = 1)2:GroupBA
##                                  -0.7649
## optimizer (nloptwrap) convergence code: 0 (OK) ; 0 optimizer warnings; 1 lme4 warnings
```

Within group Effect sizes whodasS_Ftot

contrast	effect.size	SE	df	lower.CL	upper.CL
0 TAU y0 - 78 TAU y0	0.06	0.11	127.05	−0.17	0.28
0 TAU y0 - 444 TAU y0	0.24	0.12	95.72	0.00	0.48
0 BA y0 - 78 BA y0	0.45	0.10	127.77	0.25	0.65
0 BA y0 - 444 BA y0	0.48	0.11	98.22	0.26	0.71

y ~ bs(dy, knots = 78, degree = 1) * Group + y0 + (bs(dy, knots = 78, degree = 1) + 0 | sid) sigma used for effect sizes: 9.327

1.3.2 Between group effect sizes

We looked at the effect sizes between groups by contrasting the mean WHODAS-scores between groups at post-treatment and 12-month follow-up. Used sigma=9.326677 (SD of baseline WHODAS-score)

Between group Effect sizes whodasS_Ftot

contrast	effect.size	SE	df	lower.CL	upper.CL
78 TAU - 78 BA	0.39	0.15	127.05	0.09	0.69
444 TAU - 444 BA	0.24	0.16	95.72	−0.08	0.56

y ~ bs(dy, knots = 78, degree = 1) * Group + y0 + (bs(dy, knots = 78, degree = 1) + 0 | sid) sigma used for effect sizes: 9.327

2 Sensitivity analyses

2.1 medication as covariate (as a proxy of physical illness at baseline)

We added number of medicine for phyisical illnesses, as prescribed by a doctor at baseline as a proxy for physical health at baseline,to determine whether physical health explains functional improvement.

Linear mixed model fit by REML. t-tests use Satterthwaite's method

y ~ bs(dy, knots = 78, degree = 1) * Group * fysmed_totaal + y0 + (bs(dy, knots = 78, degree = 1) + 0 | sid)

Number of obs: 367, nsid, 157

Fixed effects Anova

Coef	Sum Sq	Mean Sq	NumDF	DenDF	F value	Pr(>F)	sig
bs(dy, knots = 78, degree = 1)	0.0516	0.0258	2	102	2.46	0.090	
Group	0.0114	0.0114	1	149	1.09	0.299	
fysmed_totaal	0.00562	0.00562	1	149	0.537	0.465	
y0	11,500	11,500	1	149	1,100,000	0.000	*
bs(dy, knots = 78, degree = 1):Group	0.0506	0.0253	2	102	2.42	0.094	
bs(dy, knots = 78, degree = 1):fysmed_totaal	0.00195	0.000976	2	103	0.0932	0.911	
Group:fysmed_totaal	0.00704	0.00704	1	149	0.672	0.414	
bs(dy, knots = 78, degree = 1):Group:fysmed_totaal	0.0119	0.00596	2	103	0.569	0.568	

* p <= 0.05

Fixed effects:

Coef	Estimate	Std. Error	df	t value	Pr(>|t|)	sig
(Intercept)	0.0258	0.0268	149	0.961	0.338	
bs(dy, knots = 78, degree = 1)1	0.629	2.03	123	0.310	0.757	
bs(dy, knots = 78, degree = 1)2	−2.75	2.84	84.3	−0.967	0.336	
GroupBA	−0.0333	0.0320	149	−1.04	0.299	
fysmed_totaal	−0.00438	0.00442	149	−0.990	0.324	
y0	1.00	0.000954	149	1,050	0.000	*
bs(dy, knots = 78, degree = 1)1:GroupBA	−5.75	2.63	123	−2.18	0.031	*
bs(dy, knots = 78, degree = 1)2:GroupBA	−0.641	3.55	84.1	−0.181	0.857	
bs(dy, knots = 78, degree = 1)1:fysmed_totaal	−0.174	0.359	123	−0.484	0.629	
bs(dy, knots = 78, degree = 1)2:fysmed_totaal	0.0482	0.492	84.1	0.0980	0.922	
GroupBA:fysmed_totaal	0.00449	0.00547	149	0.820	0.414	
bs(dy, knots = 78, degree = 1)1:GroupBA:fysmed_totaal	0.358	0.465	123	0.769	0.443	
bs(dy, knots = 78, degree = 1)2:GroupBA:fysmed_totaal	−0.360	0.630	84.1	−0.571	0.570	

* p <= 0.05

Random effects

Group	Name	Variance	Std.Dev.
sid	bs(dy, knots = 78, degree = 1)1	59.06632	7.68546
sid	bs(dy, knots = 78, degree = 1)2	76.61356	8.75292
Residual	NA	0.01047	0.10232

2.2 Age analyses

We added age as a covariate to our final model,to determine whether age explains functional improvement.

Characteristic	TAU, N = 64[1]	BA, N = 94[1]	p-value[2]
Age	74 (6)	76 (7)	0.2

[1] Mean (SD)
[2] Wilcoxon rank sum test

Linear mixed model fit by REML. t-tests use Satterthwaite's method

y ~ bs(dy, knots = 78, degree = 1) * Group * Age + y0 + (bs(dy, knots = 78, degree = 1) + 0 | sid)

Number of obs: 372, nsid, 159

Fixed effects Anova

Coef	Sum Sq	Mean Sq	NumDF	DenDF	F value	Pr(>F)	sig
bs(dy, knots = 78, degree = 1)	0.00281	0.00140	2	106	0.139	0.871	
Group	0.0327	0.0327	1	152	3.22	0.075	
Age	0.000727	0.000727	1	152	0.0718	0.789	
y0	13,500	13,500	1	152	1,330,000	0.000	*
bs(dy, knots = 78, degree = 1):Group	0.0159	0.00797	2	106	0.787	0.458	
bs(dy, knots = 78, degree = 1):Age	0.000981	0.000491	2	106	0.0484	0.953	
Group:Age	0.0310	0.0310	1	152	3.06	0.082	
bs(dy, knots = 78, degree = 1):Group:Age	0.0196	0.00980	2	106	0.967	0.383	

* p <= 0.05

Fixed effects:

Coef	Estimate	Std. Error	df	t value	Pr(>|t|)	sig
(Intercept)	0.202	0.156	152	1.30	0.197	
bs(dy, knots = 78, degree = 1)1	−9.63	13.8	124	−0.697	0.487	
bs(dy, knots = 78, degree = 1)2	−17.1	17.5	84.8	−0.974	0.333	
GroupBA	−0.349	0.195	152	−1.80	0.075	
Age	−0.00262	0.00209	152	−1.25	0.213	
y0	1.00	0.000868	152	1,150	0.000	*
bs(dy, knots = 78, degree = 1)1:GroupBA	10.2	17.3	124	0.592	0.555	
bs(dy, knots = 78, degree = 1)2:GroupBA	29.7	24.7	85.8	1.20	0.232	
bs(dy, knots = 78, degree = 1)1:Age	0.124	0.189	124	0.659	0.511	
bs(dy, knots = 78, degree = 1)2:Age	0.194	0.238	84.8	0.815	0.417	
GroupBA:Age	0.00454	0.00259	152	1.75	0.082	
bs(dy, knots = 78, degree = 1)1:GroupBA:Age	−0.187	0.232	124	−0.804	0.423	
bs(dy, knots = 78, degree = 1)2:GroupBA:Age	−0.423	0.332	85.8	−1.28	0.206	

* p <= 0.05

Random effects

Group	Name	Variance	Std.Dev.
sid	bs(dy, knots = 78, degree = 1)1	61.11325	7.81750
sid	bs(dy, knots = 78, degree = 1)2	77.74637	8.81739
Residual	NA	0.01013	0.10066

2.3 Education analyses

We added Education (ranging from 1 (no education) to 6 (university degree)) to our final model to determine whether education explains functional improvement.

Education per group

Characteristic	TAU, N = 64[1]	BA, N = 94[1]	p-value[2]
Education	2.91 (1.41)	3.07 (1.55)	0.6
Unknown	0	2	

[1] Mean (SD)
[2] Wilcoxon rank sum test

Linear mixed model fit by REML. t-tests use Satterthwaite's method

y ~ bs(dy, knots = 78, degree = 1) * Group * Education + y0 + (bs(dy, knots = 78, degree = 1) + 0 | sid)

Number of obs: 366, nsid, 156

Fixed effects Anova

Coef	Sum Sq	Mean Sq	NumDF	DenDF	F value	Pr(>F)	sig
bs(dy, knots = 78, degree = 1)	0.0634	0.0317	2	102	3.03	0.053	*
Group	0.00122	0.00122	1	150	0.116	0.734	
Education	0.00133	0.00133	1	150	0.127	0.722	
y0	12,800	12,800	1	150	1,230,000	0.000	*
bs(dy, knots = 78, degree = 1):Group	0.0326	0.0163	2	102	1.56	0.215	
bs(dy, knots = 78, degree = 1):Education	0.0108	0.00541	2	102	0.517	0.598	
Group:Education	0.0000819	0.0000819	1	150	0.00783	0.930	
bs(dy, knots = 78, degree = 1):Group:Education	0.00482	0.00241	2	102	0.230	0.795	

* p <= 0.05

Fixed effects:

Coef	Estimate	Std. Error	df	t value	Pr(>|t|)	sig
(Intercept)	0.00165	0.0350	150	0.0470	0.963	
bs(dy, knots = 78, degree = 1)1	−0.853	2.59	121	−0.329	0.742	
bs(dy, knots = 78, degree = 1)2	−1.78	3.23	83.5	−0.551	0.583	
GroupBA	−0.0129	0.0379	150	−0.341	0.734	
Education	0.00157	0.00925	150	0.170	0.865	
y0	1.00	0.000903	150	1,110	0.000	*
bs(dy, knots = 78, degree = 1)1:GroupBA	−5.81	3.33	121	−1.74	0.084	
bs(dy, knots = 78, degree = 1)2:GroupBA	−2.92	4.39	84.2	−0.666	0.507	
bs(dy, knots = 78, degree = 1)1:Education	0.105	0.791	121	0.133	0.894	
bs(dy, knots = 78, degree = 1)2:Education	−0.379	1.03	84.0	−0.366	0.715	
GroupBA:Education	0.00102	0.0115	150	0.0885	0.930	
bs(dy, knots = 78, degree = 1)1:GroupBA:Education	0.662	0.993	121	0.666	0.506	
bs(dy, knots = 78, degree = 1)2:GroupBA:Education	0.366	1.32	84.2	0.278	0.782	

* p <= 0.05

Random effects

Group	Name	Variance	Std.Dev.
sid	bs(dy, knots = 78, degree = 1)1	60.70117	7.79110
sid	bs(dy, knots = 78, degree = 1)2	78.90226	8.88269
Residual	NA	0.01047	0.10230

2.4 Sex analyses

We added sex as a covariate to our analyses, to determine whether sex explains functional improvement.

Linear mixed model fit by REML. t-tests use Satterthwaite's method

y ~ bs(dy, knots = 78, degree = 1) * Group * Sex + y0 + (bs(dy, knots = 78, degree = 1) + 0 | sid)

Number of obs: 374, nsid, 160

Fixed effects Anova

Coef	Sum Sq	Mean Sq	NumDF	DenDF	F value	Pr(>F)	sig
bs(dy, knots = 78, degree = 1)	0.0710	0.0355	2	106	3.46	0.035	*
Group	0.00564	0.00564	1	152	0.549	0.460	
Sex	0.0107	0.0107	1	152	1.04	0.308	
y0	13,200	13,200	1	152	1,290,000	0.000	*
bs(dy, knots = 78, degree = 1):Group	0.0270	0.0135	2	106	1.32	0.273	
bs(dy, knots = 78, degree = 1):Sex	0.0154	0.00769	2	107	0.750	0.475	
Group:Sex	0.00318	0.00318	1	152	0.310	0.579	
bs(dy, knots = 78, degree = 1):Group:Sex	0.00734	0.00367	2	107	0.358	0.700	

* p <= 0.05

Fixed effects:

Coef	Estimate	Std. Error	df	t value	Pr(>|t|)	sig
(Intercept)	0.0480	0.0429	152	1.12	0.265	
bs(dy, knots = 78, degree = 1)1	−1.15	3.26	125	−0.352	0.725	
bs(dy, knots = 78, degree = 1)2	−4.18	4.17	86.1	−1.00	0.318	
GroupBA	−0.0374	0.0504	152	−0.741	0.460	
Sex	−0.0266	0.0256	152	−1.04	0.300	
y0	1.00	0.000882	152	1,130	0.000	*
bs(dy, knots = 78, degree = 1)1:GroupBA	−6.85	4.23	126	−1.62	0.108	
bs(dy, knots = 78, degree = 1)2:GroupBA	−2.52	5.62	86.7	−0.449	0.655	
bs(dy, knots = 78, degree = 1)1:Sex	0.425	2.11	125	0.201	0.841	
bs(dy, knots = 78, degree = 1)2:Sex	0.978	2.85	86.6	0.343	0.732	
GroupBA:Sex	0.0186	0.0335	152	0.556	0.579	
bs(dy, knots = 78, degree = 1)1:GroupBA:Sex	2.37	2.80	125	0.845	0.399	
bs(dy, knots = 78, degree = 1)2:GroupBA:Sex	0.598	3.87	86.9	0.155	0.878	

* p <= 0.05

Random effects

Group	Name	Variance	Std.Dev.
sid	bs(dy, knots = 78, degree = 1)1	59.84227	7.73578
sid	bs(dy, knots = 78, degree = 1)2	77.84558	8.82301
Residual	NA	0.01026	0.10130

2.5 MoCA analyses

We added MoCA score as a covariate to the analyses, to determine whether cognitive decline explains functional improvement.

Linear mixed model fit by REML. t-tests use Satterthwaite's method

y ~ bs(dy, knots = 78, degree = 1) * Group * moca_score + y0 + (bs(dy, knots = 78, degree = 1) + 0 | sid)

Number of obs: 363, nsid, 154

Fixed effects Anova

Coef	Sum Sq	Mean Sq	NumDF	DenDF	F value	Pr(>F)	sig
bs(dy, knots = 78, degree = 1)	0.0478	0.0239	2	101	2.31	0.104	
Group	0.0481	0.0481	1	147	4.66	0.033	*
moca_score	0.0155	0.0155	1	147	1.50	0.222	
y0	12,900	12,900	1	147	1,250,000	0.000	*
bs(dy, knots = 78, degree = 1):Group	0.0320	0.0160	2	101	1.55	0.217	
bs(dy, knots = 78, degree = 1):moca_score	0.0303	0.0151	2	101	1.47	0.235	
Group:moca_score	0.0510	0.0510	1	147	4.95	0.028	*
bs(dy, knots = 78, degree = 1):Group:moca_score	0.0403	0.0201	2	101	1.95	0.147	

* p <= 0.05

Fixed effects:

Coef	Estimate	Std. Error	df	t value	Pr(>|t|)	sig
(Intercept)	−0.297	0.145	147	−2.05	0.042	*
bs(dy, knots = 78, degree = 1)1	−27.0	12.5	121	−2.16	0.033	*
bs(dy, knots = 78, degree = 1)2	−24.9	15.8	83.3	−1.58	0.119	
GroupBA	0.377	0.175	147	2.16	0.033	*
moca_score	0.0117	0.00549	147	2.12	0.035	*
y0	1.00	0.000895	147	1,120	0.000	*
bs(dy, knots = 78, degree = 1)1:GroupBA	25.4	15.4	121	1.65	0.101	
bs(dy, knots = 78, degree = 1)2:GroupBA	19.6	20.6	84.0	0.954	0.343	
bs(dy, knots = 78, degree = 1)1:moca_score	0.997	0.475	121	2.10	0.038	*
bs(dy, knots = 78, degree = 1)2:moca_score	0.843	0.599	83.3	1.41	0.163	
GroupBA:moca_score	−0.0150	0.00675	147	−2.22	0.028	*
bs(dy, knots = 78, degree = 1)1:GroupBA:moca_score	−1.10	0.590	121	−1.87	0.064	
bs(dy, knots = 78, degree = 1)2:GroupBA:moca_score	−0.813	0.783	84.0	−1.04	0.302	

* p <= 0.05

Random effects

Group	Name	Variance	Std.Dev.
sid	bs(dy, knots = 78, degree = 1)1	59.29770	7.70050
sid	bs(dy, knots = 78, degree = 1)2	77.66983	8.81305
Residual	NA	0.01031	0.10156

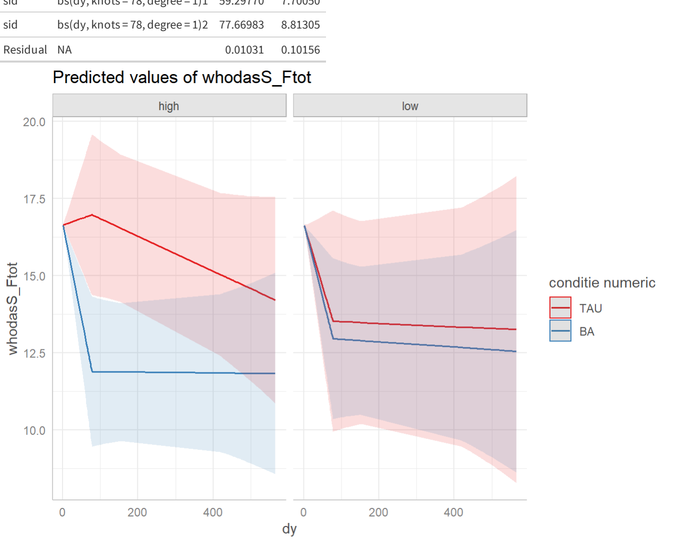

Supplement: Janssen et al. supplementary material [file S0924933823024331sup001.pdf]
